# The ANFIS-RSM based multi-objective optimization and modelling of ultrasound-assisted extraction of polyphenols from jamun fruit *(Syzygium cumini)*

**DOI:** 10.1016/j.ultsonch.2025.107227

**Published:** 2025-01-12

**Authors:** Mohammad Ganje, Somayyeh Gharibi, Fatemeh Nejatpour, Maryam Deilamipour, Kimia Goshadehrou, Sahra Saberyan, Gholamreza Abdi

**Affiliations:** a Department of Agriculture, Minab Higher Education Center, University of Hormozgan, Bandar Abbas, Iran; b Persian Gulf Marine Biotechnology Research Center, The Persian Gulf Biomedical Sciences Research Institute, Bushehr University of Medical Sciences, Bushehr, 75147, Iran; c Department of Nutrition, Faculty of Health and Nutrition Sciences, Yasuj University of Medical Sciences, Yasuj, Iran; d Faculty of Agriculture and Natural Resources, Khuzestan Science and Research Branch, Islamic Azad University, Ahvaz, Iran; e Department of Microbiology, Faculty of Biological Sciences, Alzahra University, Tehran, Iran; f Department of Biotechnology, Persian Gulf Research Institute, Persian Gulf University, Bushehr, Iran

**Keywords:** Polyphenol extraction, Botanic source, Optimization, Modelling, Ultrasound-assisted

## Abstract

Given their potential as natural substitutes for artificial additives and their health advantages, the extraction of bioactive substances like polyphenols from plant sources is becoming more and more significant. Nevertheless, it is still difficult to achieve effective extraction with minimal time and energy. In order to optimize polyphenol extraction from ripe jamun fruit pulp, including traditional and ultrasound-assisted methods, this study assessed the prediction power of response surface methodology (RSM) and adaptive neuro-fuzzy inference systems (ANFIS). It examined how temperature, process time, solvent type, and extraction method affected the yield of extracted polyphenols. Analysis of variance (ANOVA) indicated that solvent type (F-value = 292.15) was the most significant factor influencing polyphenol extraction. Numerical optimization identified optimal conditions for maximizing phenolic compound extraction: a process temperature of 45 °C, a duration of 65 min under ultrasound, using methanol as the solvent (desirability of 0.935 and a realization rate of 95 % of the maximum possible). Imposing minimum temperature and process time conditions will yield the same optimal process parameters as before, achieving 89 % of the maximum possible while significantly reducing the process time from 65 min to just 5 min (desirability 0.953). For each of the six process-solver conditions, optimal ANFIS models were determined by analyzing the number and type of input membership functions, the output membership function, and the selected optimization and defuzzification methods, based on the highest correlation between actual and predicted data, along with the lowest error rates. Statistical analysis confirmed the effectiveness of both RSM and ANFIS in modeling polyphenol extraction from ripe jamun fruit. Error indices demonstrated that ANFIS (R^2^ = 0.8490–0.9989) outperformed RSM (R^2^ = 0.9265) in predictive capability, underscoring the relative superiority of ANFIS.

## 1 Introduction

Antioxidants are essential for scavenging free radicals and avoiding oxidative stress, which is connected to a number of chronic illnesses, including cancer, heart disease, and neurological problems. Antioxidants from natural sources, such polyphenols, have drawn a lot of interest because of their possible health advantages and capacity to take the place of artificial additives in food and medicine. Research on the extraction and use of these bioactive substances has become crucial, especially for the development of effective, environmentally friendly techniques. Using cutting-edge methods like ultrasound-assisted extraction, this study aims to maximize the extraction of polyphenols from jamun fruit (Syzygium cumini), a fruit that is high in antioxidants.The Myrtaceae family includes the tropical fruit jamon, whose mature fruit has a pinkish flesh and a purple-black skin [1]. This fruit's fleshy portion is edible, tastes somewhat sweet, and has long been used to treat diseases including diabetes and diarrhea [2]. Its numerous bioactive components, including minerals, vitamins, anthocyanins, phenolic compounds, and acids like gallic, tannic, and oxalig, provide it antibacterial, antifungal, and anti-inflammatory qualities. Products including fruit juice, wine, jam, and its dry powder are made from this fruit. Phenolic substances, including epicatechin, protocatechuic acid, vanillin, syringic acid, ellagic acid, gallic acid, caffeic acid, pyrogallol, ferulic acid, catechin, and vanillic acid, are an important phytochemical in this product [3].

High-value materials with organoleptic, preservation, and therapeutic qualities can be produced by extracting and purifying important plant components [4]. Plant extracts and essential oils are increasingly being used as alternatives to synthetic materials, particularly in light of the notable increase in the usage of flavorings, colorants, preservatives, and other chemical and synthetic components [5]. Given the structural sensitivities and common instabilities that exist in these bioactive compounds [6], achieving extraction methods that maximize yield, minimize damage, and consume the least energy and time has always been of particular importance.

Due to cavitation effects that break down cell walls and facilitate mass transfer, ultrasound-assisted extraction (UAE) has a number of benefits over traditional techniques, such as lower energy usage, quicker processing times, and higher yields of bioactive chemicals [7], [8]. This technique reduces deterioration during extraction, making it especially useful for delicate bioactive substances like polyphenols. An effective method for simulating intricate nonlinear processes, such as the extraction of bioactive compounds, is the Adaptive Neuro-Fuzzy Inference System (ANFIS), a hybrid model that combines neural networks with fuzzy logic. It has been demonstrated that ANFIS performs better in terms of optimization and prediction accuracy than conventional statistical techniques [8], [9].

The ultrasonic approach has been widely employed in recent years to extract a variety of bioactive chemicals from a wide range of plant sources. The effectiveness of this technique in improving the extraction process has been highlighted in nearly every instance. Many bioactive chemicals have been extracted from a wide range of plant sources using the ultrasonic approach in recent years. The effectiveness of this technique in improving the extraction process has been highlighted in nearly every instance [10], [11], [12]. phenolic compounds are among the compounds extracted using ultrasonic-assisted extraction from different plant sources; extraction of polyphenols from Ligustrum robustum [13], Chinese propolis [14], spent coffee grounds [15], edible flower Clitoria ternatea [16], Phyllanthi Fructus [17], pine needles [18], green tea leaves [19] and pomegranate peels [20] can be mentioned as examples. In this method, the extraction process occurs more quickly and easily due to the phenomenon of cavitation and the intense stresses created in plant tissues [21]. Applying an ultrasonic bath and mixing it with an appropriate solvent can speed up the extraction process and improve the yield's quality and quantity while using less time and energy. It is possible to precisely identify the process's effective elements by optimizing and modeling these novel extraction techniques, which will produce output that is both higher quality and predictable.

Using a Box-Behnken design, Abdallah et al. (2024) improved the Ultrasound-Assisted Extraction (UAE) to extract flavonoids and polyphenols from Citrus aurantium peels [22]. A solid-to-liquid ratio of 2.4 g/100 mL, 80 % acetone, and a sonication duration of 34.7 min were the ideal extraction parameters. In comparison to traditional maceration, the UAE approach produced reduced tannin content (19.85 mg/g), greater yields of polyphenols (33.76 mg/g), and flavonoids (75.50 mg/g). Superior antioxidant activity was also demonstrated by the UAE extract. These results demonstrate that UAE is a quick and effective method for removing bioactive substances from citrus aurantium peel.

González et al., (2022) evaluated the extraction of anthocyanins from blackcurrant using Ultrasound-Assisted Extraction (UAE) and Enzyme-Assisted Extraction (EAE). Key factors were found via the Plackett–Burman design, and the solvent composition (ethanol in water) had the greatest impact. Five minutes was found to be the ideal extraction time for UAE and ten minutes for EAE, according to optimization utilizing the Box-Behnken design. Both techniques effectively applied to goods made from blackcurrants for quality control analysis and produced comparable anthocyanin extraction findings [23].

In order to improve conditions for the extraction of polyphenol-rich fractions with high antioxidant capacity and oils with good yield and oxidation stability, Piasecka et al., (2024) examined the ultrasound-assisted extraction (UAE) of polyphenols and oils from blackcurrant and redcurrant pomaces [24]. Optimal UAE settings were established using response surface methodology (RSM) based on ultrasonic amplitude (30 %-80 %) and duration (2–10 min). The ideal circumstances for polyphenols were 3 min and 51 % amplitude for blackcurrant and 11 min and 91 % amplitude for redcurrant. 99 % amplitude and 11.5 min for blackcurrant and 96 % amplitude and 12 min for redcurrant were the ideal conditions for oils. RSM is a useful method for streamlining the UAE process for both polyphenol extracts and seed oils, as demonstrated by the fact that the actual findings matched the predictions.

In contrast to conventional maceration, this study assessed the phenolic components extracted from dried blackcurrant pomace (DBP) using ultrasound-assisted extraction (UAE). In comparison to maceration, UAE at 256 W for 25 min improved the yield by 40 % and the concentration of total monomeric anthocyanins by 33 %, total phenolic content by 16 %, and radical scavenging activity by 26 %. Higher concentrations of anthocyanins, hydroxycinnamic acid, flavonols, myricetin, and quercetin were seen in ultrasound-treated DBP, along with porous, honeycomb-like structures. The findings demonstrate that ultrasonically is a more effective extraction technique than conventional maceration [25].

The Adaptive Neuro-Fuzzy Inference System (ANFIS) and Response Surface Methodology (RSM) are two popular approaches for modeling and improving nonlinear processes, such the extraction of bioactive chemicals from plant sources [26]. RSM is a statistical method that optimizes and examines how various factors affect a specific response [27]. With this approach, a response surface is modeled using experimental data, and polynomial equations are created to predict the system's behavior in various areas. RSM provides optimization curves and efficiently detects variable interactions [28]. ANFIS being a hybrid model of neural networks and fuzzy logic, is very suitable for modeling complex and nonlinear processes [29]. This system simultaneously utilizes the learning power of neural networks [30] and the interpretive capability of fuzzy logic [31], resulting in a powerful tool for predicting outputs of systems like extraction. Several studies have focused on modeling and optimizing the extraction process using these methods, including optimization and analysis of extraction of bioactive polyphenols from garcinia indica [32], mimosa pudica [33], taro (colocasia esculenta) [34], solanum torvum [35], vitis vinifera seeds [36], asparagus racemosus roots [37] and etc.

According to what has been stated, it is crucial to substitute plant-based bioactive substances with synthetic additions. At the same time, choosing and refining extraction techniques can significantly help in acquiring these molecules because of their sensitivity and instability. The goal of this study is to develop mathematical and neuro-fuzzy structures that can optimize the effects of important parameters and predict and track the extraction trend throughout the process, given that prior research has demonstrated the presence of multiple phenolic compounds in the jamun plant.

## 2 Materials and methods

### 2.1 Plant material

The ripe Jamun fruit (Syzygium cumini) was picked from Persian Gulf University's garden trees in Bushehr, Iran. The fruit pieces were dried in an oven set at 40 °C for 48 h after the pulp was separated. The beneficial components were preserved while consistent moisture removal was guaranteed by this carefully regulated drying procedure. After that, a Retsch SK1 laboratory cross-beater mill fitted with a 0.8 mm screen was used to smash the dried fruit pieces.

### 2.2 Ultrasound-Assisted and conventional extraction

Polyphenols were extracted using water, 70 % methanol, and 70 % ethanol. Sigma Aldrich provided the 96 % ethanol and methanol, and a GFL 2004 distillation machine was used on-site to distill the water. To guarantee quick and complete moistening of the jamuns while keeping the solid/liquid (S/L) ratio at 1:10, 5 g of powdered jamun were combined with 50 ml of any solvent in 250 ml reaction containers. The reaction containers were submerged in the Sonorex RK 100H ultrasonic thermostatic bath, manufactured by Bandelin Electronic GmbH & Co. KG in Berlin, Germany. It operated at a frequency of 35 kHz and had a power output of 320 W. The chamber had a capacity of 5 L, and the ultrasound intensity was approximately 0.064 W/cm^2^.

**Fig. 1:**
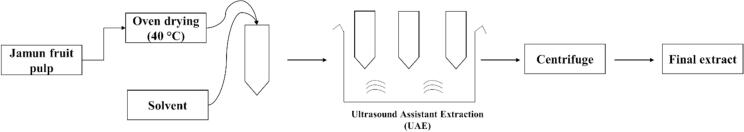
Schematic of the extraction system in this study.

For varied lengths of time (5, 25, 45, and 65 min), the extraction procedure was carried out at three constant temperatures: 45, 55, and 65 °C. After the extraction procedure, the extracts were centrifuged for four minutes at 4000 rpm in a Hettich Rotofix 32 centrifuge. After that, the resultant supernatant was collected and set aside for further examination. In order to compare the results with and without the usage of ultrasound technology, the aforementioned procedure was conducted without it. The extraction schematic is shown in Fig. 1.

### 2.3 Determination of total phenolic content (TPC)

The Folin–Ciocalteu technique was used to evaluate TPC [38]; the colorimetric interaction between the reagent and the sample is the basis for this technique. One milliliter of the extract was mixed with five milliliters of distilled water, two milliliters of sodium carbonate (Na2CO3) at a concentration of 100 g/L, and half a milliliter of Folin-Ciocalteu reagent. After that, this combination was let to stand for 90 min at room temperature in the dark. To determine the TPC, the absorbance at a wavelength of 765 nm was measured using a UV–vis spectrophotometer (GBS Avanta). A calibration curve created using a standard gallic acid solution was used to compare the results. In milligrams per gram of samples, the TPC was reported using gallic acid equivalents (mg GAE g^−1^).

### 2.4 Response surface methodology

Response surface methodology was used to analyze and optimize polyphenol extraction by estimating the linear, interaction, and quadratic effects of variables on responses [39], [40]. Design Expert software version 13′s Historical Data function was used to build an experimental design in full factorial form. Two qualitative variables—solvent type (S or X3) and process type (P or X4)—as well as two quantitative variables—ultrasound process temperature (represented as T in the real form or as X1 in the coded form) and time (t or X2)—were included in this investigation (Table 1). Preliminary test results were used to identify each factor's variance range. Interestingly, the ripe jamun pulp powder's particle size and solid/liquid (S/L) ratio were not taken into account, despite the fact that smaller particle sizes are known to boost extraction yields and the extraction of bioactive chemicals [41]. The response variable for the experimental designs was the total phenolic content (TPC). Process optimization was performed to obtain the best combination of process variables for optimum extraction efficiency.

**Table 1:** Levels of independent variables used for RSM* modelling and optimization.

Independent variables		Units	Uncoded values			
Uncoded	coded					
Temperature	X_1_	°C	45	55	65	
Time	X_2_	min	5	25	45	65
Solvent Type	X_3_	−	Methanol (M)	Ethanol (E)	Water (W)	
Process Type	X_4_	−	Ultrasound (U)	Bain-marie (B)		

*Study Type: Response Surface**,** Subtype: Randomized**,** Design Model: Quadratic.

The purpose of this study is to determine how extraction factors impact the rate at which polyphenols are extracted from ripe jamun pulp. The link between input and output variables was described using an extended response surface model, which is a second-order polynomial equation (Eq. (1) with interaction components.

(1) $$Y=\beta_{0}+\sum_{i=1}^{n}\beta_{i}X_{i}+\sum_{i=1}^{n-1}\sum_{j=2}^{n}\beta_{\mathrm{ij}}X_{i}X_{j}+\sum_{i=1}^{n}\beta_{\mathrm{ii}}X_{i}^{2}$$

Here Y refers to the predicted total phenolic content. β_0_ is the constant coefficient, whereas β_i_, β_ii_, and β_ij_ are the coefficients of linear, quadratic, and interaction effects, respectively. X_i_ and X_j_ represent independent variables and ε depicts the error. Dependent variable values (total phenolic content) were determined in triplicate, and the using a response surface analysis.

### 2.5 Adaptive neuro-fuzzy inference system (ANFIS)

ANFIS uses fuzzy logic and neural networks to analyze complicated nonlinear systems efficiently and quickly for patterns. Commonly utilized values (Table 2) were evaluated to determine the best settings for the ANFIS system under six solvent-process scenarios. The goal was to increase correlation and reduce error between model outputs and laboratory data. The correctness of these parameters in developing the ANFIS systems was assessed using MATLAB 2016a. When assessing these systems, the least difficult ones—those with the fewest input membership functions and the most straightforward parameters for others—were given precedence.

**Table 2:** List of the used parameters in ANFIS training.

Input membership function type	Number of input membership function for any input	Output membership function type	Number of epochs	Method of optimization	andMethod	Defuzzification method
Trimf	2	Constant	3	Backpropa	Min	Wtaver
Trapmf	3	Linear	4	Hybrid	Prod	Wtsum
Gbellmf	4	−	5	−	−	−
Gaussmf	5	−	6	−	−	−

### 2.6 Model appraisal studies

The predictive accuracy of RSM and ANFIS in capturing the system's nonlinear nature was evaluated using error indices, consistent with methods employed by other researchers [42], [43]. The four error function models used in this study—coefficient of determination (R^2^), absolute average relative error (AARE), root mean square error (RMSE), and standard deviation (SD)—are detailed in Eqs. (2), (3), (4), (5) respectively.

(2) $$R^{2}=1-\frac{\sum_{i=1}^{N}{\left(y_{\exp.(i)}-y_{\mathrm{pred}.(i)}\right)}^{2}}{\sum_{i=1}^{N}{\left(y_{\mathrm{pred}.(i)}-y_{\exp.ave}\right)}^{2}}$$

(3) $$\mathrm{AARE}=\frac{1}{N}\sum_{i=1}^{N}\left(\left|\frac{y_{\exp.(i)}-y_{\mathrm{pred}.(i)}}{y_{\exp.(i)}}\right|\right)$$

(4) $$\mathrm{RMSE}=\sqrt{\frac{1}{N-1}\sum_{i=1}^{N}{{(y}_{\mathrm{pred}.(i)}-y_{\exp.(i)})}^{2}}$$

(5) $$\mathrm{SD}=\sqrt{\frac{1}{N-1}\sum_{i=1}^{N}{\left(\left|\frac{y_{\exp.(i)}-y_{\mathrm{pred}.\left(i\right)}}{y_{\exp.(i)}}\right|-AARE\right)}^{2}}$$

## 3 Result and desiccation

### 3.1 RSM modelling

The direct, square, and interaction effects of each independent variable (process temperature and time, solvent, and process type) on the quantity of extracted phenolic chemicals were examined using the response surface technique. The analysis of variance table (Table 3) was utilized for this purpose as well as to identify the parameters that significantly impact the extraction rate of phenolic compounds. ANOVA-estimated p-value and F-value levels are crucial metrics for assessing the applicability and precision of the RSM model. All parameters with a p-value < 5 % (p-value < 0.05) were considered significant model terms since the likelihood of the p-value was examined at a confidence level > 95 %. X1, X3, X4, X1X3, and X1X4 were therefore important model terms (Table 3). Also, the ANOVA result in Table 3 excluded X2, X1X2, X2X3, X2X4, X3X4, X12, and X22 since they were insignificant model terms. The relevance of each term in the model was evaluated using the corresponding magnitudes of F-values in addition to the p-values. Within the model, the F index is an important indicator of impact and relevance; a higher value signifies more significance [44]. Considering the information given in Table 3, a model F-values of 43.32 was obtained, illustrating the significance of model, relative to pure error. Additionally, the predicted R-squared (0.9265) and the adjusted R-squared (09051) were in reasonable agreement. According to Onu et al., [45], a model is satisfactorily reproducible if the magnitude of difference between the R-squared and adjusted R-squared is not greater than 0.2.

**Table 3:** Analysis of variance for the model for extraction of total phenolic compounds.

Source	Sum of Squares	df	Mean Square	F-value	p-value
Model	857.61	16	53.60	43.32	< 0.0001
X_1_-Temperature	15.36	1	15.36	12.41	0.0009
X_2_-Time	0.6704	1	0.6704	0.5418	0.4648
X_3_-Solvent type	722.99	2	361.50	292.15	< 0.0001
X_4_-Process type	55.49	1	55.49	44.84	< 0.0001
X_1_X_2_	0.6765	1	0.6765	0.5467	0.4628
X_1_X_3_	53.64	2	26.82	21.67	< 0.0001
X_1_X_4_	5.07	1	5.07	4.10	0.0477
X_2_X_3_	0.4593	2	0.2296	0.1856	0.8311
X_2_X_4_	0.2338	1	0.2338	0.1890	0.6655
X_3_X_4_	1.01	2	0.5029	0.4064	0.6680
X_1_^2^	1.98	1	1.98	1.60	0.2110
X_2_^2^	0.0284	1	0.0284	0.0229	0.8802
Residual	68.05	55	1.24		
Cor Total	925.66	71			
R^2^	0.9265	Adjusted R^2^	0.9051	Predicted R^2^	0.8748
Adeq Precision	20.92	Std. Dev.	1.11	C.V. %	15.15

According to Olatunji et al., [46] and Ohale et al., [47], an adequacy precision (APR) greater than 4.0 is required for a high precision model capable of effectively navigating the design space. In this study, the value of this index was 20.93, which is much higher than 4 and indicates the high accuracy of the created model.

**Fig. 2:**
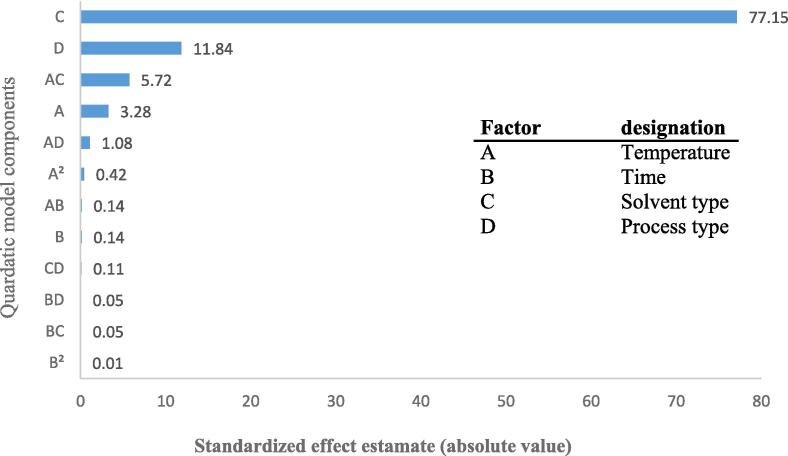
Pareto chart showing the standardized effect of independent variables and their interaction on the phenolic compound extraction.

Eq. (6) was used to determine each term's impact on the quadratic model's overall performance, and the pareto effect plot that was produced is shown in Fig. 2. From the biggest to the smallest effect, the Pareto plot displays the absolute values of the standardized effects [48]. The length of each bar in the chart indicates the standardized effect of that factor on the response [49]. Fig. 2 demonstrates that the solvent type had the greatest impact on the extraction of phenolic compounds, followed by the process type, the relationship between temperature and solvent type, temperature and other factors.

(6) $$P_{i}=\left[\frac{F_{i}}{\sum F_{i}}\right]\times 100$$

In this equation, $P_{i}$ represents the absolute value of the standardized effect estimate, while $F_{i}$ denotes the F-value index for each component of the quadratic model.

### 3.2 Effect of process parameters

#### 3.2.1 Effect of liner process parameters

**Fig. 3:**
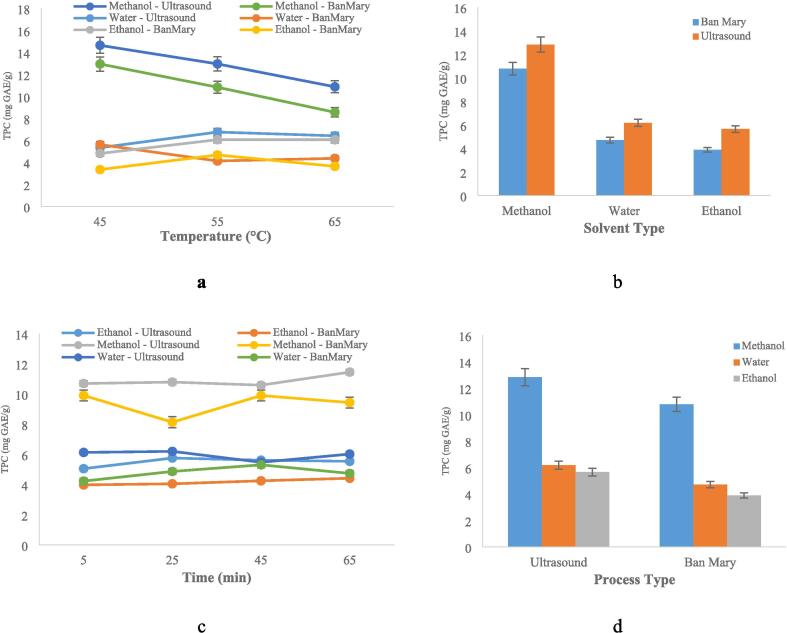
Effect of different extraction variables (temperature (a), solvent type (b), time (c), and process type (d)) on the polyphenol compound extraction.

Methanol significantly increased the effect of process temperature on the extraction of phenolic compounds (Fig. 3a). Raising the temperature had little impact on ethanol and water. Increased phenolic component breakdown brought on by higher temperatures during methanol extraction caused the extracted quantity to converge with that obtained using ethanol and water. This implies that phenolic compounds, which cannot be extracted using less polar solvents, are extremely sensitive to temperature when extracted using methanol, with many of them deteriorating between 55 and 65 °C. Compared to polar compounds, which have stronger dipole–dipole connections, non-polar or less polar compounds probably degrade more at higher temperatures because of their weaker intermolecular linkages [50].

The graph in Fig. 3b shows that methanol extracted significantly more phenolic compounds than water and ethanol. This enhanced extraction capability is due to methanol's greater polarity, which allows for the extraction of non-polar and semi-polar phenolic compounds [51]. The solubility of polyphenols is influenced by factors such as hydroxyl group presence, molecular size, and hydrocarbon chain length, highlighting the importance of solvent type in extraction [52]. Solvent is an important consideration in extraction, as it highly correlates to the affinity of the compound targeted, polarity and polar groups in fact a material will dissolve in a solvent of the same polarity [53], [54]. In a comparison of polar and non-polar solvents (water, methanol, ethyl acetate, and hexane) for extracting phenolic compounds from Strobilanthes crispus and Clinacanthus nutans, the highest yield was found with methanol, which is a polar solvent [55]. In another study, Wuryatmo et al., [56] found that extracting polyphenolic compounds from lemongrass using a polar solvent (ethanol) was more effective than using semi-polar (ethyl acetate) and non-polar (hexane) solvents.

In terms of process time, Fig. 3c shows that regardless of extraction time, quick extraction was made possible by the jamun fruit pulp's soft texture and low structural resistance as well as the comparatively high extraction temperatures. When comparing the Ben-Marie approach with ultrasound-assisted extraction, the low structural resistance was also visible (Fig. 3d). The difference was not substantial, despite the fact that ultrasound therapy was somewhat more successful. The comparable outcomes from both techniques are probably explained by the extraction mechanisms—cavitation in ultrasonic and mild heating in Ben-Marie—as well as the different solubility and thermal resistance of the chemicals. Even while the ultrasonic approach extracted more phenolic chemicals, cavitation could have caused considerable deterioration. Pan et al., [57] and d'Alessandro et al., [58] have reported that the ultrasound method, compared to extraction without it, can lead to higher levels of phenolic compounds. But, at the same time, they have also considered the possibility of destruction and/or oxidation of these compounds due to this process. Similar results have been emphasized in the study by Medina-Torres et al., [59].

#### 3.2.2 Effect of combined process parameters

**Fig. 4:**
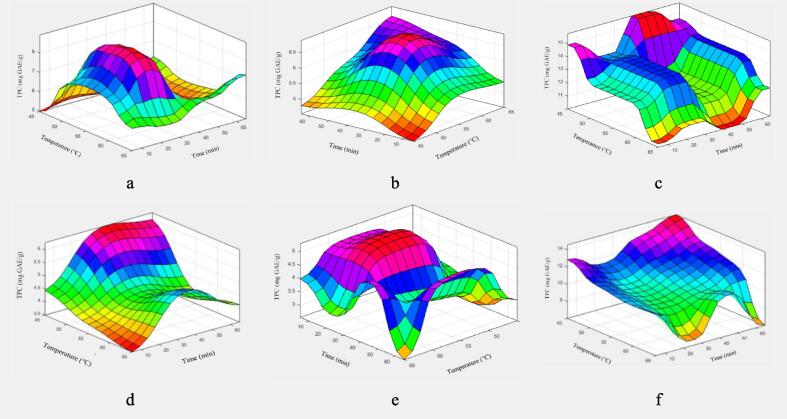
The simultaneous effect of process temperature and time on phenolic compound extraction for the Water – Ultrasound (a), Ethanol – Ultrasound (b), Methanol – Ultrasound (c), Water − Bain-marie (d), Ethanol − Bain-marie (e) and Methanol − Bain-marie (f).

Fig. 4a to c provide a thorough presentation of the combined effects of temperature and process duration on different solvents, both with and without the application of ultrasound. A close look at these illustrations and the matching color-coded tables shows notable variations that demonstrate the major impact of solvent type and the sort of processing method used. In particular, the observed trends and the maximum and lowest data points in Fig. 4a to c, which depict the ultrasonic process using water, ethanol, and methanol as solvents, exhibit significant variance even though they were all exposed to the same temperature and time circumstances. This variance can be interpreted as a clear indication of each solvent's significant effects and highlights the distinct differences in their behaviors under the same experimental conditions.

Additionally, the three graphs connected to the Ben Marie process in Fig. 4d to f may be seen in a similar manner. A thorough comparison of graph pairs that use different processing techniques but concentrate on the same solvents makes it easier to see the significant influence that processing technique selection has. Fig. 4a and d, for example, show findings from the Ben Marie method and the ultrasonic process, respectively, and both deal with the methanol solvent. The differences in outcomes between these two methods are not only evident but also emphasize the crucial role that the processing technique plays in the overall effectiveness and results obtained from the solvents. A study by Beaudor et al. (2023) revealed that ultrasound-assisted extraction recovers over 83 % of polyphenols from spent coffee grounds, surpassing conventional extraction's 64 % efficiency and cutting energy consumption by over 50 %. This demonstrates a 33 % improvement in polyphenol recovery efficiency with ultrasound [15]. In Porto et al. (2013), ultrasound-assisted extraction produced a grape seed oil yield of 14 % w/w in just 30 min, demonstrating significantly greater efficiency compared to a 6-hour Soxhlet extraction [60].

Accurate 3D diagrams can identify the temperature and time limits for maximum extraction of phenolic compounds across six solvent-process conditions. The highest extraction for water occurred at 55 to 60 °C under ultrasound for 20 to 30 min and at 45 °C for 35 to 45 min in a bain-marie. For ethanol, the optimal conditions were 55 to 60 °C and 30 to 45 min (ultrasound) and 50 to 60 °C for 20 to 30 min (bain-marie). Maximum extraction using methanol was also achieved under ultrasound at 45 °C for 40 to 50 min and in a bain-marie at 45 °C for 65 min.

### 3.3 Multi-objective optimization

To find the ideal mix of process variables for maximum extraction efficiency, process optimization was carried out. The inability to account for interactions among variables and the requirement for large amounts of data are two major disadvantages of optimization strategies that examine the impact of one component at a time while holding the rest constant. Response surface approach was used to overcome these problems [61]. Its goal is to attain the optimal response by concurrently optimizing the values of operational variables. To optimize the extraction process, this study considered two conditions. The first involved defining all input factors within a range without limits. The second prioritized low temperature and time for extraction optimization (Table 4). In the first condition, a temperature of 45 °C, a time of 65 min, methanol solvent, and ultrasound extraction yielded 14.95 mg GAE/g of phenolic compounds with a desirability of 0.935. In the second condition, the process parameters were minimized to 45 °C and 5 min (Table 4), resulting in a 0.97 mg decrease in phenolic compounds compared to the first condition but achieving a desirability of 0.953. Despite the significant reduction in processing time, the second condition appears more favorable. The maximum phenolic compound yield in this study was 15.77 mg GAE/g, indicating that the first and second conditions achieved 95 % and 89 % of the maximum extraction, respectively.

**Table 4:** Numerical optimization of poly phenol extraction.

Name		Criteria and Constraints				Solution	Response	Desirability
		Goal		Lower Limit	Upper Limit			
condition (1)	Temperature	in range		45	65	45	14.95	0.935
	Time	in range		5	65	65		
	Solvent Type	in range		M	E	M		
	Process Type	in range		B	U	U		
condition (2)	Temperature	minimize		45	65	45	13.98	0.953
	Time	minimize		5	65	5		
	Solvent Type	in range		M	E	M		
	Process Type	in range		B	U	U		

### 3.4 ANFIS modelling

Six distinct neural-fuzzy models were created for this investigation because of the different process circumstances. Each model corresponds to distinct extraction methods: ethanol under ultrasound and without ultrasound, methanol under ultrasound and without, and water under ultrasound and without. With two inputs (temperature and process time) and one output (TPC), all six models made use of Sogno's fuzzy structure. In order to choose the best systems, the models—which were based on the parameters given in Table 2—were assessed by comparing output values with the actual data and evaluating statistical indicators such as the R2, RMSE, AARE, and SD (Table 5). All of the chosen ideal models showed adequate accuracy, with correlation coefficients ranging from 0.85 to 0.999 and RMSE, AARE, and SD less than 0.980, 0.0485, and 0.1148, respectively.

**Table 5:** Framework and training parameters for optimum ANFIS models.

ANFIS parameters	Ethanol − Ultrasound	Ethanol − Bain-marie	Methanol − Ultrasound	Methanol − Bain-marie	Water − Ultrasound	Water − Bain-marie
Number of nodes	35	53	53	53	53	35
Number of linear parameters	9	16	16	16	16	9
Number Of nonlinear parameters	12	24	32	24	24	12
Total number of parameters	21	40	48	40	40	21
Number of fuzzy rules	9	16	16	16	16	9
Number of input membership function	3	4	4	4	4	3
Membership function	Gaussmf	Gbllmf	Trampf	Gbllmf	Gbllmf	Gaussmf
Output membership function	Constant	Constant	Constant	Constant	Constant	Constant
Method of optimization	Hybrid	Hybrid	Hybrid	Hybrid	Hybrid	Hybrid
and Method	'Prod'	'Prod'	'Prod'	'Min'	'Min'	'Prod'
Defuzzification method	'Wtaver'	'Wtaver'	'Wtaver'	'Wtaver'	'Wtaver'	'Wtaver'
Number of epochs	3	3	3	3	3	3

### 3.5 Model appraisal analysis

**Fig. 5:**
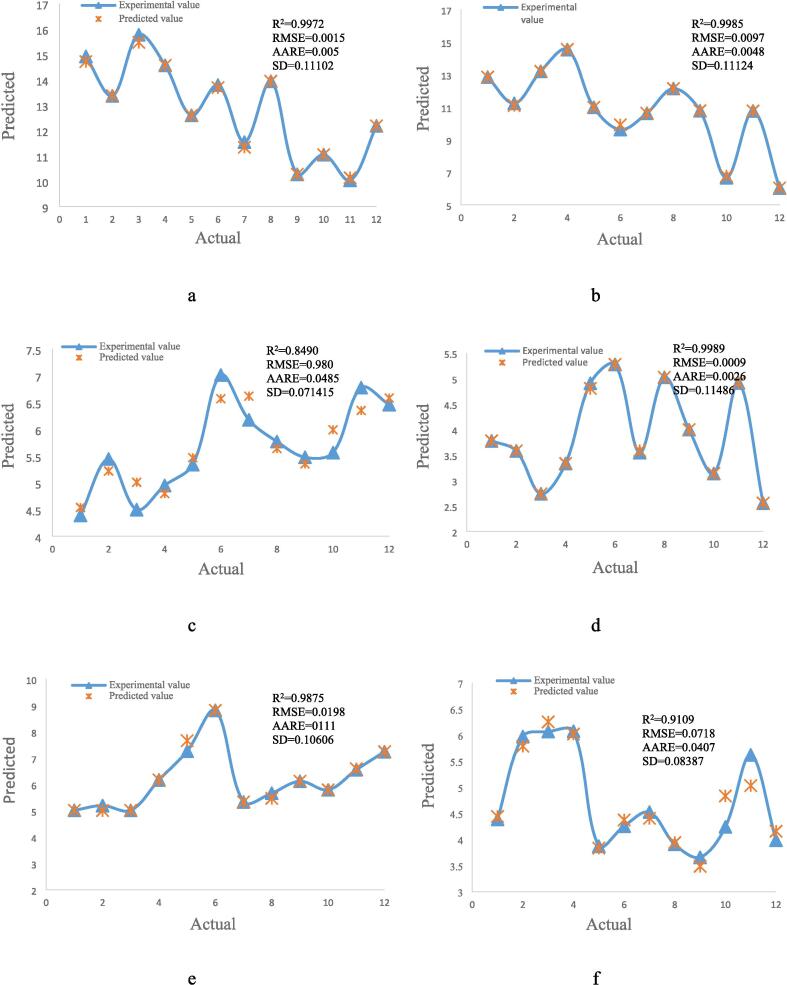

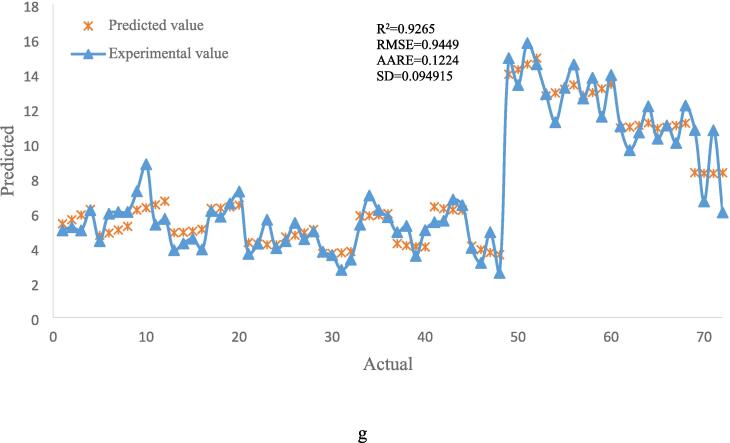
Actual vs predicted plots for ANFIS (Methanol – Ultrasound (a), Methanol − Bain-marie (b), Ethanol – Ultrasound (c), Ethanol − Bain-marie (d), Water – Ultrasound (e) and Water − Bain-marie (f)), and RSM (g) modeling.

Predicted values were plotted against actual values, and statistical markers including the R^2^, RMSE, AARE, and SD were used to assess the accuracy of the optimum models that were produced using the RSM and ANFIS approaches (Fig. 5). The efficacy of RSM and ANFIS models in forecasting the extraction process is evidenced by their strong R^2^ values. The mean relative error between the experimental and anticipated data is roughly represented by AARE, which stands for data deviation. The best RSM model's value of 0.1224 is much larger than the greatest AARE for the ANFIS models (extraction with ethanol solvent under ultrasound), which is 0.0485. In extraction using ethanol solvent without ultrasound, where ANFIS is around 50 times more precise, this benefit is much more pronounced. Kunjiappan et al. (2024) utilized RSM and ANFIS to optimize polyphenol extraction from grape seeds. They found that RSM offered a solid quadratic model, but ANFIS demonstrated superior prediction accuracy, indicating its potential for complex optimization tasks in this area [36]. Farzaneh et al. (2017) assessed models using ANFIS, finding those with an R^2^ higher than 0.99 to be effective for modeling sesame seed extraction [62].

### 3.6 Conclusion

The RSM and ANFIS methods were used in this work to improve the UAE method for jamun fruit polyphenolic component extraction. The kind of solvent had the most effect on the total phenolic content (TPC), followed by the extraction method, temperature, and duration, according to a factorial ANOVA. Two distinct sets of circumstances were used to conduct the optimization procedure. The first condition underwent ultrasonic treatment at 45 °C and methanol extraction for 65 min, yielding 14.95 mg of phenolic compounds (with a desirability score of 0.935). 9.5 % and 89 % of the maximum yield (15.77 mg GAE/g) were obtained in the second condition, which had an extraction duration of 5 min at 45 °C and produced 14.98 mg of phenolics. This illustrates how simultaneous optimization may effectively increase extraction efficiency. Furthermore, six neural-fuzzy models were created with and without ultrasound utilizing a variety of extraction techniques using ethanol, methanol, and water. The best model structure for each scenario was identified using statistical analysis based on R^2^, RMSE, AARE, and SD. The higher performance of ANFIS in predicting TPC extraction was proven by comparisons between the RSM and ANFIS models, which consistently showed fewer errors than RSM. An effective strategy for improving polyphenolic extraction procedures is the use of ANFIS as a predictive tool, which has the potential to significantly lower operating costs, reduce processing time, and improve the quality of the finished product.

## 4 Ethical review

This study does not involve any human or animal testing.

## 5 Funding statement

This research did not receive any specific grant from funding agencies in the public, commercial, or not-for-profit sectors.

## CRediT authorship contribution statement

**Mohammad Ganje:** Writing – original draft, Validation, Supervision, Software, Project administration, Methodology. **Somayyeh Gharibi:** Writing – review & editing, Methodology. **Fatemeh Nejatpour:** Writing – review & editing, Funding acquisition. **Maryam Deilamipour:** Writing – review & editing, Investigation, Data curation. **Kimia Goshadehrou:** Investigation, Funding acquisition, Data curation. **Sahra Saberyan:** Writing – review & editing, Methodology, Investigation. **Gholamreza Abdi:** Writing – review & editing, Data curation, Conceptualization.

## 6 Data availability

The data will be made available upon reasonable request.

## Declaration of competing interest

The authors declare that they have no known competing financial interests or personal relationships that could have appeared to influence the work reported in this paper.
